# Effects of protein or amino-acid supplementation on the physical growth of young children in low-income countries

**DOI:** 10.1093/nutrit/nux027

**Published:** 2017-07-29

**Authors:** Joanne E Arsenault, Kenneth H Brown

**Affiliations:** 1 Program in International and Community Nutrition, University of California, Davis, USA; 2 Bill and Melinda Gates Foundation, Seattle, Washington, USA

**Keywords:** child growth, low-income countries, protein, stunting, supplementation

## Abstract

Child growth stunting is common in low-income countries, possibly due to insufficient protein intakes. Most previous studies have concluded that children’s protein intakes are adequate in relation to estimated requirements, but these studies did not consider issues of protein digestibility and effects of infection on dietary protein utilization. Using an alternative approach to assess the possible role of protein inadequacy in children’s growth restriction, the results of 18 intervention trials in which supplementary protein or amino acids were provided to children ages 6–35 months and growth outcomes were reviewed. Eight studies conducted in hospitalized children recovering from acute malnutrition found that the recommended protein intake levels for healthy children supported normal growth rates, but higher intakes were needed for accelerated rates of “catch-up” growth. Ten community-based studies did not demonstrate a consistent benefit of supplemental protein on children’s growth. However, weaknesses in the study designs limit the conclusions that can be drawn from these studies, and additional appropriately designed trials are needed to answer this question definitively. Recommendations for optimizing future study designs are provided herein.

## INTRODUCTION

Approximately one-third of children aged <5 years worldwide have stunted linear growth, defined as a height-for-age *Z* score (HAZ) <−2 standard deviations (SDs) with respect to international growth standards. Stunting is associated with an increased risk of child mortality, infectious disease morbidity, impaired neurocognitive development, and metabolic diseases in later life; it is, therefore, of critical concern for public health.^1^ Stunting is attributable to multiple factors, including small-for-gestational-age and preterm births, inadequate dietary quantity and quality, morbidity from infections, and poor child-care practices.^1^^,^^2^ Among the multiple nutritional factors that affect growth, adequate protein is needed for tissue synthesis in addition to the amount required for maintenance of normal body functions.

Research on dietary protein adequacy in low-income countries indicates the vast majority of young children consume more protein than specified in published estimates of protein requirements,^3–5^ resulting in the common understanding that growth restriction is not due to protein deficiency. Therefore, other nutritional and nonnutritional causes of growth restriction have been investigated. However, recent controversies regarding the methods for assessing dietary protein adequacy create uncertainty about the possible role of dietary protein inadequacy in growth restriction. For example, a report published by the Food and Agriculture Organization of the United Nations (FAO) in 2013 suggested that the true availability of amino acids from the diet may be less than currently assumed when estimates of protein digestibility are based on fecal nitrogen excretion (so-called “fecal digestibility”) rather than individual amino-acid digestibility and uptake in the ileum (ie, “ileal digestibility”).^6^ However, little information is currently available on ileal digestibility for individual amino acids, so it is not possible to adjust protein intake accordingly. In addition, previous studies of protein intake adequacy did not use currently recommended dietary assessment methods, such as (1) including multiple days of individual observations to establish the population distribution of usual intakes, (2) applying the estimated average requirement cut-point method to assess prevalence of low intakes below requirements, and (3) adjusting for protein quality. Furthermore, these studies were unable to adjust for the effects of infections on protein utilization. Because of these uncertainties in the interpretation of studies of dietary protein adequacy, an alternative approach to assessing the possible role of protein in growth restriction is to examine the impact of supplemental protein on children’s growth. This article briefly reviews current estimates of young children’s protein requirements and then summarizes the results of previously published intervention trials in which additional protein or selected amino acids were provided to young children at risk of stunting.

## ESTIMATED PROTEIN REQUIREMENTS OF YOUNG CHILDREN

The protein requirements of infants and children are defined as the minimum intake that will allow nitrogen equilibrium during energy balance (for maintenance) plus the amount needed for deposition of new tissues (for growth).^7^ Protein requirements are expressed per kilogram body weight (BW). The World Health Organization (WHO), FAO, and the United Nations University (UNU)^7^ estimated daily protein requirements of infants and children at 0.66 grams per kilogram BW for maintenance, plus age-related requirements for growth, which decrease progressively as growth velocity slows in older children (Table 1^7–9^). The US Institute of Medicine published similar estimates to those issued by WHO/FAO/UNU,^9^ with the amino-acid requirements of children expressed per kilogram BW and decreasing with age (Table 2^6^^,^^7^).

**Table 1:** Estimates of protein requirements of infants and children from the World Health Organization/Food and Agriculture Organization of the United Nations/United Nations University and Institute of Medicine

Age	Maintenance, g/kg body weight per day	Growth, g/kg body weight per day	Average total requirement, g/kg body weight per day	Recommended intake (average requirement +2 SD), g/kg body weight per day	Median body weight, kg	Recommended intake (average requirement +2 SD), g/d
World Health Organization/Food and Agriculture Organization of the United Nations/United Nations University^a^						
0.5 y	0.66	0.46	1.12	1.31	7.6	10.0
1 y	0.66	0.29	0.95	1.14	9.3	10.5
1.5 y	0.66	0.19	0.85	1.03	10.6	10.9
2 y	0.66	0.13	0.79	0.97	11.9	11.5
3 y	0.66	0.07	0.73	0.90	14.1	12.7
4 y	0.66	0.03	0.69	0.86	16.2	13.9
5 y	0.66	0.03	0.69	0.85	18.3	15.5
Institute of Medicine^b^						
7–12 mo	0.69	0.31	1.00	1.20	9	11
1–3 y	0.69	0.18	0.87	1.05	12	13
4–8 y	0.69	0.07	0.76	0.95	20	19

^a^Data from Table 33a in World Health Organization/Food and Agriculture Organization of the United Nations/United Nations University (2007).^7^ The protein requirement for growth for 5 years was modified due to a presumed error (0.06 g/kg) in the report. Values were only provided on a gram per kilogram body weight basis. The recommended intake level of protein per day was calculated using median body weights from World Health Organization child growth standards for specified age.^8^

^b^Data from Institute of Medicine (2002).^9^

**Table 2:** Amino-acid requirements of children and scoring patterns for protein quality assessment

Age, y	Amino acid								
	Histidine	Isoleucine	Leucine	Lysine	Sulfur-amino acids	Aromatic-amino acids	Threonine	Tryptophan	Valine
Requirement (mg/kg body weight)^a^									
0.5	22	36	73	64	31	59	34	9.5	49
1–2	15	27	54	45	22	40	23	6.4	36
3–10	12	23	44	35	17	30	18	4.8	29
Scoring pattern (mg/g protein requirement)^b^									
0.5 ^b^	20	32	66	57	27	52	31	8.5	43
1–2	18	31	63	52	25	46	27	7.4	42
3–10	16	31	61	48	23	41	25	6.6	40

^a^Amino acid requirement data are from Table 36 in World Health Organization/Food and Agriculture Organization of the United Nations/United Nations University (2007).^7^ The requirements for the sulfur amino acids were modified due to calculation errors identified and reported in Table 3 in Food and Agriculture Organization of the United Nations (2013).^6^

^b^The 2013 Food and Agriculture Organization of the United Nations report^6^ recommends the scoring pattern for infants aged 0.5 years be used for young children (6 months to 3 years).

Dietary protein available to the body and utilizable for protein metabolism depends on the digestibility of the protein and its amino-acid composition. The proteins in breast milk and animal-source foods are considered to be of “good quality” because they are highly digestible and comprised of more than adequate amounts of all of the essential amino acids. Plant-based foods tend to have poorer quality protein because their proteins are less digestible and contain lower amounts of some essential amino acids, particularly lysine and sometimes tryptophan (in cereals) and sulfur-containing amino acids (in legumes). The quality of dietary protein intake can be assessed by calculating a protein digestibility-corrected amino acid score (PDCAAS), which is the product of the digestibility (proportion of protein digested and absorbed) and the lowest ratio of each amino acid in the diet (or food) to the amino-acid composition of a reference protein.^6^ The reference scoring pattern of amino acids used in calculating the PDCAAS is presented in Table 2, and sample calculations of the PDCAAS for single foods are depicted in Supplementary Table S1 (see Table S1 in the Supporting Information online). The PDCAAS can also be used to characterize whole diets by calculating the sum of digested amino acids from all foods and comparing it with the scoring pattern, as illustrated by the example in Supplementary Table S2 (see Table S2 in the Supporting Information online). The PDCAAS can be used to adjust the total dietary protein intake to estimate the amount of protein in the diet that is available to the body—that is, “available” protein = total protein intake × PDCAAS.^7^ The amount of PDCAAS-adjusted (or available) protein provided by a food or a mixed diet is not usually described in publications of intervention trials, so in cases where the available protein intakes could be calculated from the studies reviewed here, that information has been provided using the methods presented in Supplementary Tables S1 and S2 (see Tables S1 and S2 in the Supporting Information online).

## REVIEWS OF INTERVENTION TRIALS

The results of intervention studies that provided supplementary protein or amino acids to children aged 6–35 months and reported growth outcomes are reviewed below. Growth outcomes may include changes in weight or length in absolute terms or in relation to established growth indices, such as *Z* scores, with respect to reference values. To address the question of whether inadequate protein intake is a cause of growth restriction in children, the most useful studies are ones that provided supplemental protein to randomly selected groups of children and compared their growth with the growth of children who did not receive supplemental protein. Ideally, such randomized intervention trials should be conducted in populations with a high risk of stunted growth and among children within the age range in which stunting occurs. The usual baseline protein intakes in the study populations should be suspected to be inadequate or marginally adequate in relation to estimated requirements, and information on total protein intake should be collected during the study period to monitor adherence to the intervention and potential displacement of the usual diet. The intervention groups should differ only in relation to their protein or amino-acid intakes, and energy and other nutrients required for growth should be provided in adequate amounts. The duration of the intervention should be sufficient to assess linear growth. Finally, the sample size should be adequate to examine potential modifying factors, such as initial anthropometric status, diet, and morbidity. These considerations were taken into account when examining the studies reviewed in this article, although few of the available studies successfully addressed even a subset of these issues.

## METHODS

A PubMed search was conducted in February 2015 using the following search terms: [(children or infants) AND (protein or amino acids) AND (supplementation or intervention) AND growth]. Additional searches were conducted for each essential amino acid using the following search terms (example for lysine): [(lysine) AND (children or infants) AND growth AND (supplementation or intervention or fortification or fortified)].

The titles and abstracts of all articles were reviewed for appropriateness. Additional references were identified from articles found during the search. Studies with children aged 6–35 months were included. The majority of studies identified were excluded due to ≥1 of the following reasons: (1) study not an intervention trial, (2) age of children outside of target age range, (3) children had clinical conditions other than recovery from malnutrition that could affect their growth, and (4) interventions differed by other nutrients in addition to protein.

Eighteen studies were identified for inclusion in this review. Information on the studies was extracted by 1 author (J.E.A.) into an Excel spreadsheet with the following information, if available: author, year, research site, type of protein or amino acid, study participants, study design, intervention description, baseline anthropometry, growth outcomes, other outcomes, statistical analysis method, diet information, and study limitations. Eight of the studies were conducted in hospitalized children recovering from acute malnutrition, and the following interventions were used: lysine-supplemented cereals (n = 4), quality protein maize (QPM) (n = 1), potato as the protein source (n = 1), and varied levels of energy from cow’s milk protein (n = 2). Ten studies were community-based trials that examined the following types of intervention: QPM (n = 7), glutamine (n = 1), and lipid-based supplements that varied protein source or amount (n = 2). Results of this review are presented according to the 2 categories of inpatient and community-based trials.

## RESULTS AND DISCUSSION OF PROTEIN SUPPLEMENTATION TRIALS

### Inpatient studies conducted in children recovering from acute malnutrition

The 8 studies in this category were all conducted in Peru by the same set of investigators working with children who had been hospitalized previously for treatment of severe, acute malnutrition. Four small, short-term studies were designed to determine whether supplemental lysine added to cereal-based diets would increase weight gain and linear growth.^10–13^ Among the remaining studies, 1 tested QPM^14^ and 1 tested potato^15^ as a protein source, and 2 evaluated the adequacy of different levels of protein intake for children recovering from malnutrition.^16^^,^^17^ Details of the studies are summarized in Table 3^10–17^ Additional details on the dietary intakes and estimated PDCAAS of the diets are provided in Table 4^10–14^ for studies that provided sufficient information.

**Table 3:** Overview of inpatient studies of protein supplementation and growth of young children recovering from malnutrition

Reference	Country	Participants	Design	Type of protein supplementation	Intervention	Duration	Growth outcomes	Additional information
Graham et al. (1969)^10^	Peru	N = 6; aged 11–24 mo	Crossover	Lysine added to wheat	All infants assigned to 5 phases of diets, the 4 wheat diets assigned in random order for 15–36 d with intervening 9-d periods of casein: (1) casein as protein source; (2) wheat as protein source (W); (3) wheat with lysine, 0.12% enrichment (WL1); (4) wheat with lysine, 0.2% enrichment (WL2); (5) wheat with lysine, 0.4% enrichment (WL3)	15–36 d	Weight gains (as percentage of the rate during the casein period) were not significantly different between W (67% ± 11.6%) and WL1 (83% ± 14.2%) Weight gains were different (*P* < 0.05) between W and WL2 (97% ± 22.7%) and W and WL3 (91% ± 15%).	Nitrogen retention (as percentage of that during casein period) was different between W and all 3 lysine groups (*P* < 0.01). Among lysine groups, the only difference was between WL1 and WL2 (*P* < 0.02).
Graham et al. (1971)^11^	Peru	N = 6; aged 10–43 mo	Crossover	Lysine added to wheat	Infants assigned to 1 of 3 wheat diets for 3–6 mo, preceded and followed by casein diets: (1) WL1; (2) WL2; (3) WL3	3–6 mo	Infants who received all 3 levels of lysine-supplemented wheat and at least 8% of energy as protein had normal growth. If protein was <8% of energy, WL2 and WL3 had better growth rates than WL1.	No statistical analysis of results Only 2 subjects per diet
Graham et al. (1981)^12^	Peru	N = 13; aged 6.4–24.5 mo	Partial crossover (phase 3 only)	Lysine added to wheat	Infants assigned to 1 of 3 diet phases: (1) wheat providing 50% of energy plus casein; (2) wheat providing 75% of energy plus oil; (3) wheat providing 75% of energy plus oil with periods with and without lysine	3 mo	Phase 1: Weight and height gains exceeded expected. Phase 2: Height gain was not as expected. Phase 3: Weight gain exceeded expected; height gains were inadequate.	No statistical analysis of results
Graham et al. (1986)^13^	Peru	N = 10; aged 7–36 mo	Crossover	Lysine added to fermented sorghum	All infants assigned to 4 9-d diet periods: (1) casein; (2) fermented sorghum (with or without lysine); (3) fermented sorghum (with or without lysine); (4) casein	36 d	Weight gain was higher on casein than sorghum diets (*P* < 0.01). Weight gain was not different between sorghum diets with and without lysine.	Nitrogen retention was lower from sorghum without lysine diet than all other diets (*P* < 0.05). Nitrogen retention was higher from final casein diet than all other diets (*P* < 0.01).
Graham et al. (1990)^14^	Peru	N = 20; aged 13–29 mo	Observational	QPM (higher in lysine and tryptophan)	QPM vs cow’s milk formula	90 d	Rate of weight gain with QPM (2.63 g/kg/d) was equal to that with cow’s milk (2.60 g/kg/d)	
Lopez de Romaña et al. (1981)^15^	Peru	N = 10; aged 6–24 mo	Not specified	Potato as protein source	Children assigned to 1 of 3 potato-based diets with added casein: (1) 50% of energy from potato, 60% protein from potato (with added casein 0.94 g calcium caseinate/100 kcal); (2) 75% energy from potato, 89% protein from potato (with added casein 0.26 g ca caseinate/100 kcal); (3) 84.2% of energy from potato, 100% of protein from potato	3 mo	Mean weight gains were above expected for age. 5 children showed rapid catch-up growth.	Small number of children did not allow comparison of diet groups. The third group was dropped due to diet too bulky for infants to consume.
MacLean and Graham (1979)^16^	Peru	N = 6; aged 4–17 mo	Crossover	Cow’s milk protein	Children received 4 levels of protein for 14 d each: (1) 4% of energy from protein; (2) 5.3% of energy from protein; (3) 6.4%–6.7% of energy from protein; (4) 8% of energy from protein	2 mo	Rate of weight gain was significantly higher on 5.3% and 6.4%–6.7% energy diets than 4%. Weight gain at 8% energy was no different from that at 6.4%–6.7% energy.	Serum albumin was not significantly different between diets.
Graham et al. (1996)^17^	Peru	N = 81; aged 6–31 mo	Randomized allocation	Cow’s milk protein	Children randomized to 3 diets varying in cow’s milk protein (whey, casein): (1) 4.7%–5.5% of energy from protein (1.2–1.4 g protein/100 kcal); (2) 5.4%–6.7% energy from protein (1.6–1.7 g protein/100 kcal); (3) 8% of energy from protein (2 g protein/100 kcal)	3 mo	There were no significant differences in weight gain or linear growth among diet groups.	

*Abbreviations:* QPM, quality protein maize; W, wheat as protein source; WL1, wheat with lysine, 0.12% enrichment; WL2, wheat with lysine, 0.2% enrichment; WL3, wheat with lysine, 0.4% enrichment.

**Table 4:** Characteristics of study diets in inpatient studies of wheat, sorghum, and maize with or without supplemental lysine among Peruvian children recovering from severe malnutrition

Reference	Diets and protein source	Energy, kcal/kg body weight	Protein, g/kg body weight	Protein, % of energy	Estimated PDCAAS,^a^ %	Estimated PDCAAS-adjusted protein, g/kg body weight^a^
Graham et al. (1969)^10^	Wheat	100–125	2	6.4–8.0	45	0.90
Graham et al. (1969, 1971)^10^^,^^11^	Wheat with 37.5 mg of lysine per gram of protein (equivalent to 0.12% enrichment)	100–125	2	6.4–8.0	63	1.26
Graham et al. (1969, 1971)^10^^,^^11^	Wheat with 44 mg of lysine per gram of protein (equivalent to 0.2% enrichment)	100–125	2	6.4–8.0	74	1.48
Graham et al. (1969, 1971)^10^^,^^11^	Wheat with 61 mg of lysine per gram of protein (equivalent to 0.4% enrichment)	100–125	2	6.4–8.0	87	1.73
Graham et al. (1981)^12^	Wheat providing 50% of energy and 77%–80% of protein plus oil and casein	100–125	2–25	8.0	68	1.20–2.20
Graham et al. (1981)^12^	Wheat providing 75% of energy and 100% of protein plus oil	110–125	3.1–3.5	11.2	45	1.40–1.80
Graham et al. (1981)^12^	Wheat providing 75% of energy plus oil with periods with and without lysine added at 0.2% enrichment	110–160	3–4.3	10.9	80	2.40–3.40
Graham et al. (1986)^13^	Fermented sorghum without lysine (6.4% of energy from protein)	145 ^b^	2.33	6.4	27	0.63
Graham et al. (1986)^13^	Fermented sorghum with lysine (6.4% of energy from protein)	147 ^b^	2.35	6.4	39	0.92
Graham et al. (1990)^14^	Quality protein maize (40 mg of lysine per gram of protein, 9.2 mg of tryptophan per gram of protein)	110	2.63	9.6	60	1.60

*Abbreviation:* PDCAAS, protein digestibility-corrected amino acid score.

^a^The PDCAAS and PDCAAS-adjusted protein were not reported in published studies but were estimated by the author of this review (J.E.A.).

^b^Energy was not reported in the published papers but was estimated by the author of this review (J.E.A.).

The 4 lysine studies conducted in Peru each used a similar crossover design with 6–13 children in each group. In general, the children were enrolled after a steady state of weight gain was achieved, and the energy level of diets was set to maintain a specified rate of weight gain, with 6.4%–8.0% of energy from protein. Three of the 4 studies examined the effects of feeding the children wheat-based diets and additional lysine, which is the most limiting amino acid in wheat. If wheat is the sole source of protein, the PDCAAS-adjusted protein is approximately 45% of the crude protein; this is due primarily to the low amount of lysine in wheat (see Supplementary Table S1 in the Supporting Information online). In the fourth trial, sorghum was the sole source of protein, with a PDCAAS of 27%.

Two studies with almost identical design were conducted to determine the amount of lysine that should be added to wheat flour for maximal improvement in biological value, measured as nitrogen retention, and rates of weight gain, while isocaloric and isonitrogenous diets were consumed for 15–36 days in the first study^10^ or 3–6 months in the second one.^11^ Six children were enrolled in each study after they had achieved a steady rate of weight gain. The children were fed a diet with casein as the unique protein source before and after randomly sequenced diets in which wheat was the only protein source, with or without 1 of 3 levels of additional lysine (see Tables 3 and 4). The wheat flour used in the diets was higher in protein content (21 g protein/100 g of flour) than ordinary wheat flour (approximately 10 g protein/100 g) and the lysine content of the flour was 26.7 mg/g of protein. Total protein intakes were designed to provide 2 g/kg BW. The authors noted that all of the diets provided less lysine than the estimated requirement of 90 mg/kg BW, but the more recently updated FAO estimate of lysine requirement is much lower, ie, 44 mg/kg BW for a child aged 1–2 years.^7^

The children’s weight gains during the 4 diet phases were expressed as a percentage of the rate achieved during the preceding and following casein periods, which was a mean of 3.8 g/kg BW/d. The mean expected rate of weight gain for children of the same chronological age is about 0.7 g/kg BW/d, and the mean expected rate of weight gain for children with the same mean weight of 7 kg (which corresponds to a chronological age of 4–6 mo) is 2–3 g/kg BW/d (based on the 2006 WHO growth standards^8^). Thus, these children were gaining weight more rapidly than expected for age or BW during all of the dietary periods and had greater protein requirements than normal-weight children.

In the first study, the mean rates of weight gain were not different for children receiving the wheat-only diet versus the wheat with 0.12% lysine enrichment diet; the former group achieved 67% of the rates of weight gain recorded during the 2 casein periods, and the latter group achieved 83% of the rate recorded during the casein periods. By contrast, rates of weight gain differed significantly (*P* < 0.05) between the wheat-only diet (67% of the rate achieved with casein) and the wheat with 0.2% lysine enrichment diet (97% of the rate achieved with casein) and the wheat with 0.4% lysine enrichment diet (91% of the rate achieved with casein). Nitrogen retention also increased during each of the 3 periods with added lysine and was similar among the 2 highest lysine-enriched wheat diets and the casein diets, which led the authors to conclude that gains in protein value were evident with the 0.12% level of lysine enrichment, further gains occurred with enrichment at the 0.2% level, and no further gain was seen at the 0.4% level.

In the second study, 2 children each received lysine-enriched wheat flour at 1 of 3 enrichment levels as their only source of dietary protein for 3–6 months.^11^ The children’s energy intakes were in the range of 100–150 kcal/kg and actual protein intakes before correcting for the PDCAAS were in the range of 1.65–2.5 g/kg BW. The authors described each child’s growth in terms of height-age or weight-age (referring to the age at which the child’s height or weight is in the 50^th^ percentile of the US growth reference) and height or weight quotient (100 × height- or weight-age/chronological age). Most of the children had height and weight ages far below their chronological age. One of the 2 children receiving wheat enriched with 0.12% lysine achieved height and weight gains exceeding gains in chronological age while receiving 8% of energy as protein (approximately 1.26 g of available protein per kg BW), indicating catch-up growth; specifically, over 4.5 months, this child progressed in height-age by 5.5 months and in weight-age by 11.5 months. The other child receiving wheat enriched with 0.12% lysine and only 6.6% of energy as protein (approximately 1.0 g of available protein/kg BW) had lower increases in height- and weight-age (2 mo) than chronological age (3 mo). Among the 2 children who received wheat enriched with 0.2% lysine, 1 child who received 8% of energy as protein (approximately 1.48 g of available protein/kg BW) had gains in height and weight age that matched the increase in chronological age (4.5 mo). The second child who received wheat enriched with 0.2% lysine, initially at 6.7% protein energy (1.85 g of available protein/kg BW) and later at 8% protein energy, had a gain in weight-age (12.5 mo) that exceeded the gain in chronological age (4.5 mo), but a gain in height-age (3.5 mo) that was slightly below the gain in chronological age (4.5 mo). The 2 children who received wheat enriched with 0.4% lysine (predominately at 8% of protein energy and 1.7–2.2 g available protein/kg BW) had growth rates that exceeded those for chronological age. The authors concluded that wheat flour enriched with 0.1%–0.4% lysine can serve as the only source of protein in the diet for several months when the diet provides at least 8% of energy as protein; however, if the diet provides 6.4%–8% of energy from protein, 0.2%–0.4% lysine enrichment would be needed.

A third study was conducted in 13 Peruvian children recovering from malnutrition to determine whether a diet providing 75% of energy and 100% of protein as wheat could support satisfactory growth.^12^ The diets provided 100–160 kcal/kg BW/d, based on levels previously shown to maintain weight gain within an acceptable range of 3–7 g/kg BW/d for recovering malnourished children. When wheat was provided as 50% of energy and 77%–80% of protein, weight and height gains exceeded the expected gains for age; the average weight gain was 546% of the median amount expected for age, and height gain was 195% of expected. When wheat was provided as 75% of energy and 100% of protein, weight gain was greater than expected for age (252%), but linear growth was less than expected for age (86%). Finally, when wheat was provided as 75% of energy in periods with or without supplemental lysine at 0.2% enrichment, the lysine supplementation produced increases in weight gain (mean 567% of gain expected for age) that exceeded those in the nonlysine periods (mean 331% of gain expected for age). The period of lysine supplementation in this study was too short to assess gains in body length, but overall, during the entire period of the third diet (most of which did not include lysine supplementation), linear growth was inadequate. The authors concluded that supplemental protein or lysine is needed for infants consuming diets in which wheat provides 75% of energy.

The fourth study reviewed examined the impact of feeding a sorghum-based diet with and without lysine to infants recovering from acute malnutrition.^13^ Ten children were each fed 4 diets over 9-day periods; the diets included a casein control diet during the first and last periods and diets of fermented sorghum flour (1 with lysine and 1 without lysine) as the source of protein during the middle 2 periods. Weight gains were significantly higher with the casein diet compared with the sorghum diets but they did not differ between the sorghum diets with and without lysine. Apparent nitrogen retention was significantly lower during the sorghum without lysine dietary period (26%) than during the sorghum with lysine dietary period (34%) or the casein periods (35% and 49%, for periods 1 and 4, respectively), suggesting that the higher weight gains during the casein periods were reflective of the increased nitrogen retention due to higher protein quality. The higher nitrogen retention in the second casein period compared with the first indicates compensation for inadequate retention during the sorghum periods. The mean intake of PDCAAS-adjusted protein was above the protein requirements for children of this age range (0.73–1.12 g protein/kg BW, depending on age) when children consumed the sorghum flour with lysine (0.92 g/kg BW) but not the sorghum flour without lysine (0.63 g/kg/BW) (Table 4).

A fifth study compared the growth rates of 20 children who were fed either QPM or cow’s milk formula for 90 days.^14^ The QPM had a lysine content of 40 mg/g protein and 9.2 mg tryptophan/g protein and an estimated PDCAAS of 60% based on the lysine requirement pattern of 57 mg/g protein. The mean intake of the children receiving QPM was 110 kcal/kg BW and 2.63 g of protein (or 1.6 g of available protein)/kg BW. The mean rate of growth was 2.63 g/kg BW/d, which was comparable to the rate achieved by children consuming cow’s milk formula (2.6 g/kg BW/d), who were consuming an estimated 2.01 g available protein/kg BW. Although the estimates of available protein from the QPM used in the study are not certain, the estimates were well above the recommended intake level for children in this age range (1.0–1.1 g/kg BW), and this study suggests that the QPM adequately supported the children’s growth.

A sixth study, which involved 10 children recovering from malnutrition in Peru included 3 isonitrogenous, isocaloric diets with varying proportions of energy and protein from potato.^15^ The initial energy intake was estimated based on the amount required during the 2 weeks preceding the start of the study to promote a weight gain of 2–4 g/kg BW/d while consuming a cow’s milk formula with presumably adequate protein content. Each of the study diets contained 2 g protein/100 kcal (8% of energy from protein) but the amounts of potato and casein as the protein sources varied (Table 3). The third diet, which contained 84% of energy and 100% of protein from potato, was subsequently dropped because the first 2 infants who received it were not able to consume it in sufficient quantities. In the 2 remaining diets, the children’s mean daily energy and protein intakes were 124 kcal/kg BW and 2.5 g/kg BW (2.15 g available protein/kg BW) for the diet with 50% energy and 60% of protein from potato, and 129 kcal/kg BW and 2.58 g/kg BW (2.11 g available protein/kg BW) for the diet with 75% of energy and 89% of protein from potato. All of the children in these 2 groups gained weight at a rate equal to or greater than expected for their age. The small number of children (and the small differences in available protein intakes) precluded the detection of any differences between the 2 diets, but the authors concluded that both diets allowed for adequate growth.

A Peruvian study of 6 infants recovering from malnutrition evaluated an isoenergetic diet with varying levels of protein from cow’s milk formula.^16^ The children received 4 different levels of protein: (1) 4% of energy, (2) 5.3% of energy, (3) 6.4%–6.7% of energy, and (4) 8% of energy. The order of administration of the diets was randomized and each diet was provided for 14 days. The rates of weight gain were significantly lower during the 4% energy diet period (2.8 g/kg/d) than during the periods of 5.3% energy (5.2 g/kg/d; *P* < 0.05) and 6.4%–6.7% energy (6.7 g/kg/d; *P* < 0.01). The mean weight gain during the 8% energy period was 6.6 g/kg/d, which is similar to that of the 6.4%–6.7% diet; however, it was not compared statistically with the 4% diet because 2 of the children did not receive the 8% energy diet. This small study suggests that weight gain was greater with diets providing 5.3% or 6.4%–6.7% energy from cow’s milk protein compared with 4%, but there was no further increase with diets providing 8% of energy as protein.

A study of 81 children recovering from malnutrition in Peru evaluated the adequacy of recommended protein intakes for young children.^17^ Children were divided into 2 length-age strata and were randomly assigned to receive 1 of 3 isocaloric diets consisting of cow’s milk formula with varying levels of protein content for 3 months. Forty-four younger children aged 5–18 months who had an initial length age of 2.5–6.4 months received 5.5%, 6.7%, or 8% of energy from protein. Thirty-seven older children aged 11–32 months with a length-age of 6.1–17.9 months received 4.7%, 6.4%, or 8% of energy from protein. The lowest protein diets were designed to meet the 1985 FAO/WHO/UNU recommended safe level of protein intake of 1.47 g/kg BW/d for children aged 3–4 months and 1.15 g/kg BW/d for children aged 9–12 months^18^. Energy content of the diets was estimated to be sufficient to support an increase in length-age of 3 months and an increase in weight to the median length-age over the course of the study. The actual mean intakes of protein per kilogram BW for the younger children were 1.7, 1.9, and 2.5 g/kg BW for the 5.5%, 6.7%, and 8% protein diets, respectively. Older children consumed 1.3, 1.6, and 2.1 g protein/kg BW for the 4.7%, 6.4%, and 8% protein diets, respectively. There were no differences between groups in weight gain or linear growth. The authors noted that the lowest protein diet in the younger age stratum may have been marginally limiting linear growth, but the small numbers of younger children in the 3 dietary groups (n = 14–15) may have prevented their ability to detect a small statistical difference from the other diets. Based on current 2007 WHO/FAO/UNU protein recommendations,^7^ which are slightly higher than the 1985 guidelines, the lowest protein energy diet for the youngest children met the recommended intake level of 1.31 g/kg BW/d. For the older children in the lowest protein cell, a protein intake of 1.1 g/d (2 SD below the mean of 1.3 g) was sufficient for adequate growth. This study demonstrates that additional protein intake above the WHO/FAO/UNU estimated requirement did not appear to provide any additional benefit for growth during recovery from malnutrition.

The studies reviewed in this section are mostly limited by their small numbers of children and the short durations of supplementation in which to assess growth, particularly linear growth. Moreover, the children were all recovering from acute malnutrition that had been serious enough to require hospitalization. Although the children were enrolled after their weight gain and other indicators of protein status were improved, it is unclear how generalizable the findings are to the growth response of more mildly undernourished or otherwise adequately nourished children with lower expected growth rates. The last study described was especially informative in that it was designed to determine the adequacy of a then newly recommended (FAO/WHO/UNU 1985)^18^ intake level of protein (4.7%–5.5% of energy, depending on age) compared with 2 higher levels for growth of children recovering from malnutrition, and all diets were isocaloric, only differing in the amount of protein from cow’s milk. Providing additional protein above the estimated requirement did not convey any additional benefit for the growth of these children. In these studies, the children experienced varying rates of “catch-up growth,” as evidenced by higher rates of weight gain than expected for their age or body size, even within recommended protein intake ranges; this suggests that recommended protein levels should support adequate growth in generally healthy children. These studies are also informative for examining the relationship between estimated available protein intakes and rates of weight gain among children recovering from malnutrition, which is discussed further below.

### Community-based trials with food-based protein or amino-acid supplementation

Ten community-based trails were examined in this review: 7 with QPM,^19–22^ 1 with glutamine,^23^ and 2 with lipid-based nutrient supplements.^24^^,^^25^ The latter 2 studies tested the difference between soy and milk protein. These studies are summarized in Table 5.^19–25^

**Table 5:** Overview of community-based studies of protein supplementation and growth of young children in low-income countries

Reference	Country	Participants	Design	Type of protein supplementation	Intervention	Duration	Growth outcomes	Additional information
Gunaratna et al. (2010)^19^	Ethiopia, Ghana, India	N = 1330; aged 4–56 mo (from 7 studies)	Meta-analysis	QPM	QPM vs CM provided as seed or dough	6–13 mo	Children consuming QPM had 12% (95%CI, 7%–18%) greater rate of weight gain than CM (*P* = 0.0002), and 9% greater (95%CI, 7%–18%) rate of height gain than CM (*P* < 0.0001)	
Akalu et al. (2010)^20^	Ethiopia	N = 151; aged 5–29 mo	Cluster randomized	QPM	Households received 15 kg of seed of QPM or CM, sufficient to sow 0.5 hectare and meet household maize needs for 1 year. Study follow-up from the start of maize consumption	13 mo	Rate of weight gain significantly higher in QPM group (*P* value not reported) WLZ decreased less in QPM group (*P* = 0.048) No differences in WAZ or LAZ changes Results for treatment groups: -QPM: gain 167 g/mo, decrease 0.09 WLZ, decrease 0.10 WAZ, decrease 0.04 LAZ -CM: gain 146 g/mo, decrease 0.36 WLZ, decrease 0.30 WAZ, decrease 0.09 LAZ	Statistical analysis appropriately assessed change in growth with control for baseline value, but small number of clusters limited the statistical power.
Singh et al. (1980)^21^	India	N = 132; aged 18–30 mo	Allocation not described	QPM	3 treatment groups fed 1 meal/d (405 kcal, 10 g protein): -QPM -CM -Skim milk powder -Control group—no meal	6 mo	No statistical analyses reported. Results for treatment groups: -QPM: gain 1.3 kg, 3.87 cm -CM: gain 1.04 kg, 2.99 cm -Milk: gain 1.2 kg, 4.22 cm -Control: gain 0.72 kg, 2.86 cm	No statistical analysis Unclear method for group assignment
Akuamoa-Boateng (2002)^22^	Ghana	N = 83; aged 4–23 mo	Double-blind random allocation	QPM	QPM or CM seeds provided to households	12 mo	No significant differences in weight or height gains Results for treatment groups: -QPM: gain 2.27 kg ( ± 0.19), 10.6 cm (±0.65) -CM: gain 2.36 kg (±0.19), 9.91 cm (±0.67)	Statistical analysis not clearly described, small sample High loss to follow-up (140 children initially) Some mixing of QPM and CM, households ran out of QPM and purchased CM
Akuamoa-Boateng (2002)^22^	Ghana	N = 78; aged 4–15 mo	Double-blind random allocation	QPM	QPM or CM dough provided to households	12 mo	Significantly greater height gain with QPM (*P* = 0.03). Results for treatment groups: -QPM: gain 2.92 kg (±0.18), 14.76 cm (±0.68) -CM: gain 2.93 kg (±0.18), 12.37 cm (±0.68)	Statistical analysis not clearly described, small sample. High loss to follow-up (120 children initially).
Akuamoa-Boateng (2002)^22^	Ghana	N = 321; aged 4–9 mo	Random allocation	QPM	QPM or CM dough (100 g/kg body weight of child) provided	12 mo	Significantly greater height gain with QPM (*P* = 0.0001); lower rate of stunting with QPM at end of study (*P* < 0.05) Results for treatment groups: -QPM: gain 2.48 kg ( ± 0.07), 13.10 cm (±0.17), 24% stunting at end -CM: gain 2.31 kg (±0.07), 12.13 cm (±0.18), 43.3% stunting at end	Statistical analysis not clearly described High loss to follow-up (422 children initially)
Akuamoa-Boateng (2002)^22^	Ghana	N = 486; aged 4–6 mo	Random allocation	QPM	Children randomized to 4 groups with and without barley malt for additional energy (50 g maize dough per kg body weight, increased to 100 g at 7 mo age): -QPM + barley malt -CM + barley malt -QPM -CM The energy density of the dough with barley was approximately 3 times greater than dough without barley	7 mo	Significantly greater weight gain with QPM + barley compared with CM + barley (*P* < 0.01); no differences between QPM and CM groups Results for treatment groups: -QPM + barley: gain 3.42 kg (±0.09), 8.07 cm (±0.30) -CM + barley: gain 2.91 kg (±0.09), 7.61 cm (±0.31) -QPM: gain 1.81 kg (±0.10), 7.42 cm (±0.33) -CM: gain 1.87 kg (±0.09), 6.91 cm (±0.32)	Statistical analysis not clearly described High loss to follow-up (600 children initially)
Williams et al. (2007)^23^	Gambia	N = 93; aged 4–10 mo	Double-blinded random allocation	Glutamine	Children randomized to 4 groups, isonitrogenous, isoenergetic mix: -Groups 1 and 2: 0.5 g/kg glutamine -Groups 3 and 4: 3–4: 0.5 g/kg nonessential amino acids (alanine, glycine, serine, asparagine) and fructose	5 mo	No significant differences in final weight, height, WAZ, or HAZ. Change variables not reported Results for treatment groups: -Glutamine: final weight 7.31 kg (±0.96), final height 69.5 cm (±3.0), final WAZ −2.69 (±0.17), final HAZ −1.73 (±0.15) -Placebo: final weight 7.33 kg (±0.97), final height 69.4 cm (±3.5), final WAZ −2.58 (±0.16), final HAZ −1.76 (±0.14)	No differences in other outcomes: intestinal permeability, plasma proteins, time with reported morbidity symptoms Preliminary study, short duration, small number of children per group
Kuusipalo et al. (2006)^24^	Malawi	N = 128; aged 6–17 mo, initial WAZ < −2	Blinded random allocation	Lipid-based supplement with milk or soy	Children randomized to 8 groups with 2 different fortified spreads (milk- or soy-based) and different doses: −25 g/d milk-based (4 g protein) −25 g/d soy-based (3 g protein) −50 g/d milk-based (8 g protein) −50 g/d soy-based (7 g protein) −75 g/d milk-based (11 g protein) −75 g/d soy-based (10 g protein) −5 g/d milk-based (1 g protein); (8) no spread	12 wk	No significant differences between milk- and soy-based groups receiving the same dose Results for treatment groups: −25 g milk: gain 0.70 kg (±0.44), 2.3 cm (±1.5); 25 g soy: gain 0.69 kg (±0.43), 2.4 cm (±1.2) −50 g milk: gain 0.83 kg (±0.37), 2.7 cm (±0.9); 50 g soy: gain 0.69 kg (±0.39), 2.8 cm (±1.2) −75 g milk: gain 0.65 kg (±0.47), 2.4 cm (±1.0) −75 g soy: gain 0.73 kg (±0.42), 2.6 cm (±0.9) −5 g milk: gain 0.56 kg (±0.23), 2.2 cm (SD missing) -None: gain 0.54 kg (±0.32), 1.7 cm (±1.3)	
Mangani et al. (2013)^25^	Malawi	N = 840; aged 6 mo (747 children completed)	Blinded random allocation	Lipid-based supplement with milk or soy	Children randomized to 4 groups with 2 different fortified spreads (milk- or soy-based): -Milk-LNS: 285 kcal, 8.2 g protein, vitamins, and minerals -Soy-LNS: 276 kcal, 7.5 g protein, vitamin, and minerals same as milk-LNS -CSB: 284 kcal, 10.4 g protein, vitamins, and minerals less than LNS groups -No supplement	12 mo	No significant differences between milk- and soy-based groups Results for treatment groups: -Milk-LNS: gain 2.53 kg (±0.78), 13.2 cm (±1.7) -Soy-LNS: gain 2.46 kg (±0.88), 13.0 cm (±2.0) -CSB: gain 2.32 kg (±0.88), 12.9 cm (±2.6) -None: gain 2.42 kg (±0.77), 13.0 cm (±2.0)	

*Abbreviations:* CM, conventional maize; CSB, corn-soy blend; LAZ, length-for-age *Z* score; LNS, lipid nutrient supplement; QPM, quality protein maize; WAZ, weight-for-age *Z* score; WLZ, weight-for-length *Z* score; HAZ, height-for-age.

*Opaque-2* varieties of maize bred for improved protein quality, specifically higher concentrations of lysine and tryptophan, were developed in the 1960s. Earlier varieties had problems with lower yields and susceptibility to pests that precluded their adoption, but improved varieties with better agronomic properties, referred to as QPM, are now available more widely, particularly in sub-Saharan Africa. The lysine and tryptophan concentrations of QPM are approximately double the concentrations in conventional maize (CM) varieties.

Seven studies that compared the growth of children consuming QPM with that of children consuming either cow’s milk formula or CM were identified. Six of these 7 studies were included in a previously published meta-analysis by Gunaratna et al.,^19^ which examined the impact of QPM versus CM on child growth. The meta-analysis included results from 7 studies conducted in Ethiopia, India, and Ghana. Children were aged <5 years, although most were <24 months of age at baseline. Quality protein maize was associated with a 12% (95% confidence interval [CI], 7%–18%) greater rate of weight gain (*P* = 0.0002) and a 9% (95%CI, 6%–15%) greater rate of height gain (*P* < 0.0001), although methodological limitations of the individual studies preclude definitive conclusions. Six of the 7 individual studies included children aged <36 months and are described in the following paragraphs.

A cluster-randomized study conducted in Ethiopia provided either QPM or CM seeds to households in sufficient quantity to meet the household’s maize needs for 1 year.^20^ The study assessed the growth of 151 children aged 5–29 months over a 13-month period. Initially, 30% of children were stunted. Over the 13 months, changes in length-for-age and weight-for-age *Z* scores did not differ between groups, although there was a marginal difference in the change in weight-for-length *Z* score (*P* = 0.048). In particular, children in the QPM group reportedly gained significantly more weight (167 g/mo) than the children in the CM group (146 g/mo), but the statistical significance of the results was not reported. Both groups of children grew an average of 0.77 cm/month. Dietary information revealed that 95% of the children were still breastfeeding at baseline and that complementary feeding consisted primarily of maize; at baseline, only 31% of the children received foods other than maize, and this was usually teff, another grain that is also limited in lysine but higher in protein (13% of energy) than maize (9% of energy). Throughout the study, even fewer of the children received other foods, particularly during the preharvest season when only 10% of children received foods other than maize. Lack of quantitative dietary information on consumption of maize, total energy, and protein intakes makes it difficult to interpret the lack of overall impact on weight-for-age or length-for-age *Z* scores.

A study in India assessed the impact of providing QPM in comparison with CM or milk on the growth of children.^21^ One hundred thirty-two children aged 18–30 months who were at least 60% of the median weight-for-age were assigned to 1 of 3 experimental groups (QPM, CM, or skim milk powder) or a control group. Children in the experimental groups received a midday meal that was designed to provide an average of 405 kcal and 10 g protein/d for 6 months. The mean weight gains over the 6 months were 1.3 kg for the QPM group, 1.2 kg for the milk group, 1.04 kg for the CM group, and 0.72 kg for the control group; and mean height gains were 4.22 cm for the milk group, 3.87 cm for the QPM group, 2.99 cm for the CM group, and 2.86 cm for the control group. The authors concluded that QPM was superior to CM and similar to skim milk for supplementary feeding of young children, but no statistical analyses were reported, so this conclusion is uncertain. Moreover, studies of the children’s total dietary intakes suggested that all were receiving 2–3-fold higher amounts of protein than their theoretical requirements, so it is difficult to understand why they would have responded to increased protein intake if the dietary data were, indeed, accurate.

A series of 4 studies conducted in Ghana were included in the meta-analysis by Gunaratna et al.^19^ and are described in a report by the Ghana Health Service.^22^ The first 2 trials were pilot or exploratory, the third trial was the main study testing the efficacy of QPM versus CM, and the fourth trial included an energy supplement in addition to the maize. The report stated that the QPM contained 70% higher amounts of lysine and tryptophan than CM.

In the first study, seed was provided to 140 households with random allocation of QPM and CM. The children were aged 4–23 months, and anthropometric measurements were taken over 12 months. Data were only available for 83 children throughout the study due to attrition. There were no differences in weight or height gains between the QPM and CM groups. The second study provided QPM and CM in the form of dough to 120 households with children aged 4–15 months for 12 months. Data were available for 78 children at the end of the study. Children receiving QPM had a mean gain in height that was 2.4 cm greater than children receiving CM (*P* = 0.03). The high attrition rate and the failure to assess the characteristics of children who did and did not complete the study undermine the usefulness of these results.

The third trial was designed to provide maize dough to 321 children aged 4–9 months for 12 months. Eighteen percent to 20% of the children were stunted at baseline. Children receiving QPM had a 1-cm greater increase in height than the children receiving CM (*P* = 0.0001). At the end of 12 months, 24% of children who received QPM were stunted compared with 43% of children who received CM (*P* < 0.05). In addition, the children who received QPM had fewer days ill from the third month of the study onward than children who received CM, although it was unclear how an illness day was defined.

Based on poor weight gains in the third trial, which was attributed to the high water content of the maize porridge, a fourth trial was designed to provide additional energy without excess bulk by adding barley malt to maize dough. Maize dough (QPM or CM) with and without barley malt was provided to 600 children aged 4–6 months for 7 months. Four hundred eighty-six children remained at the end of the study and were included in the analysis. The dough with barley malt provided approximately 3 times the energy as the dough without malt. Children receiving QPM with barley malt gained 0.5 kg more than the children receiving CM with barley malt (*P* < 0.01). There were no significant differences in length gain in children who received QPM with barley malt versus those who received CM with barley malt, nor in either weight or length gains in children who received QPM or CM without barley malt.

Some information on energy and protein intakes of the children was reported for the third and fourth trials, although not very clearly. For trial 4, only the average intakes of the *mpampa* (maize porridge with barley malt) and *koko* (maize porridge without barley malt) were presented, which provided an average of approximately 350 kcal/d and 100 kcal/d, respectively. Total protein intakes could not be estimated from the information provided. It is unclear why in the fourth trial there was no difference in growth measures between the QPM and CM without barley groups when in the third trial there was a difference. It appeared there was lower consumption of *koko* in the fourth trial (100 kcal/d vs 150 kcal/d in trial 3), which may have been due to a slightly younger age range and the duration of study in trial 4.

The QPM studies reviewed here were all conducted in areas with a high risk of stunting. Although the meta-analysis reported a positive impact of QPM on weight gain and linear growth, most of the studies cannot be considered to be of adequate quality to draw conclusions. Of the 6 studies, only 1 conducted a longitudinal analysis of growth controlling for baseline status, and that study did not find an impact on changes in length-for-age *Z* score or weight-for-age *Z* score after 1 year.^20^ Only 2 of the studies were published in peer-reviewed journals,^20^^,^^21^ and 1 did not report any statistical analyses and did not specify the method of allocation to treatment groups.^21^ The 4 studies conducted in Ghana were not peer-reviewed and had high loss to follow-up due to movement from the study area.^22^ Two of the 4 studies were small preliminary studies. As previously mentioned, the dietary protein intakes of the children were not well documented, which makes it difficult to assess the protein adequacy of the baseline diet in the studies that reported no impact of QPM. Therefore, the efficacy of QPM for child growth based on these studies remains uncertain.

A community-based trial conducted in Gambia, where disease and growth faltering are common among children, was conducted to determine if glutamine would have an impact on intestinal mucosal damage and growth of infants (Table 5).^23^ Glutamine is a nonessential amino acid, although it is thought to be “conditionally essential” during illness or injury. The double-blind, placebo-controlled, randomized trial was conducted during 5 months of the rainy season. Ninety-three infants aged 4–10 months were randomized into 1 of 4 groups: 2 of the groups received glutamine (0.5 g/kg BW daily), and 2 of the groups received an isonitrogenous, isoenergetic mix of nonessential amino acids and fructose. No differences in growth were found between the 2 groups at the end of the study. Mean weight gains were 60 g/mo and 69 g/mo, and height gains were 1.01 cm/mo and 0.95 cm/mo in the glutamine and placebo groups, respectively. Weight-for-age *Z* scores decreased during the study from an initial mean of −1.70 and −1.69 to a final mean of −2.69 and −2.58 in glutamine and placebo groups, respectively. Height-for-age *Z* score decreased during the study from an initial mean of −1.23 and −1.33 to a final mean of −1.73 and −1.76 in glutamine and placebo groups, respectively. No differences between groups were detected for intestinal permeability measures.

This trial was conducted in a population at risk of stunting. The daily provision of glutamine was nearly half of their protein requirement of 1.12 g/kg BW and the only difference in the treatment groups was the provision of glutamine versus other nonessential amino acids. There was no information provided on dietary intakes to permit assessment of the protein adequacy of the baseline diet. Nevertheless, there was no evidence of any growth impact of this particular amino acid.

Two community-based studies with a lipid-based micronutrient-fortified spread allow for the comparison of different sources of protein (milk or soy) that were added to the spread, which was otherwise equal in energy and micronutrient contents.^24^^,^^25^ The first was a preliminary study conducted in Malawi that provided the respective products to children for 12 weeks.^24^ A total of 128 children aged 6–17 months who were underweight (weight-for-age *Z* score less than −2) were randomized to 1 of 8 treatment groups. Six groups received a spread that was either milk based (dried skimmed milk) or soy based (defatted soy flour) and provided in 3 different doses (25, 50, and 75 g/d), 1 group received 5 g/d of a milk-based spread, and 1 group did not receive any supplement. Pertinent to this review are the comparison groups receiving the same amounts of either milk or soy-based spread, as they provided similar amounts of energy and protein but different types of protein varying in quality (although the PDCAAS could not be calculated from the information presented in the published report). The amounts of protein provided by the 25 g/d, 50 g/d, and 75 g/d supplements were 3–4 g, 7–8 g, and 10–11 g, respectively. No significant differences in growth were detected among comparable groups of children receiving the same doses of milk- or soy-based spreads. The study was considered preliminary, and its short duration and small numbers of children per group may have limited the ability to detect impacts on growth. At a mean initial weight of 7.5 kg, the estimated average protein requirement of the children was approximately 7–8 g/d. No baseline dietary information was reported to assess whether the children’s diets were limited in protein. It appears that the children did not benefit from the higher quality protein in the milk-based supplement or the higher amounts of protein over this short period of time.

The second study conducted in Malawi by the same researchers provided fortified spreads or corn-flour blend supplements to children for a longer duration.^25^ Eight hundred forty children aged 6 months were randomized to 1 of 4 groups: a group that received a lipid-based nutrient supplement (LNS) with dried milk (milk-LNS), a group that received LNS with soy flour (soy-LNS), a group that received a corn-soy flour blend (CSB), or a control group that received no supplement for 12 months. The comparable groups of interest here are the children who received milk-LNS or soy-LNS because they had similar nutrient contents and the supplements contained 8.2 g and 7.5 g of protein, respectively. There were no significant differences in weight gain or linear growth between children who received the milk-LNS and children who received the soy-LNS. The mean length-for-age *Z* scores were approximately −1.6 to −1.7 initially and decreased in all groups. During the 9–12-month age period, the milk-LNS group did have a significantly smaller decrease in length-for-age *Z* score ( −0.23) than the control group (−0.31; *P* = 0.03) or the CSB group (−0.38; *P* = 0.01), but not the soy-LNS group (−0.28; *P* = 0.08). At the beginning of the study, the children’s average estimated protein requirement was approximately 7.8 g/d (1.12 g/kg BW), and at the end of the study their protein requirement was approximately 8.2 g/d (0.86 g/kg BW). Therefore, the supplement alone should have met their average protein requirements, assuming all of it was consumed. No information on the children’s diets was provided, except that almost all children were receiving breast milk throughout the entire study. Although it might be possible that, if the children were consuming an amount of protein close to their requirement, they could have had some slight benefit to the higher protein quality in the milk-LNS, this seems unlikely as the actual difference in estimated available protein from the milk-LNS (95% of 8.2 g) and soy-LNS (86% of 7.5 g) is only approximately 1.3 g.

The longer study in Malawi had a suitable design, but the study population was already moderately stunted at 6 months of age, which indicates that other factors, such as prenatal influences, inadequate early diet, and morbidity, were affecting growth, and the amount and quality of the protein in the children’s diets, other than that provided by the supplement, were unknown. Despite these limitations, there is no evidence from this study to suggest that increasing the children’s protein intake above baseline levels affected their growth.

## DISCUSSION

This review identified a remarkably small number of intervention studies that specifically examined the independent impact of protein or amino-acid supplementation on child growth. Many of the studies were conducted several decades ago at small inpatient facilities among children recovering from acute malnutrition. Nevertheless, these studies offer insights into the protein needs of children experiencing accelerated growth in compensation for prior growth restriction. Their findings suggest that the presently recommended protein intake levels are sufficient to support accelerated growth and, therefore, should also support children with less severe malnutrition. The community-based studies that provided supplementary protein or amino acids did not demonstrate any consistent benefit of supplemental protein for enhancing children’s growth. A number of other recent studies have assessed the impact of food-based supplements that contained protein on children’s growth, but these supplements contained other nutrients in addition to protein, so were not suitable for assessing the independent effect of protein and were, therefore, not included in this review. Nevertheless, many of these food-based studies also failed to show improved growth among children who received supplements that included protein along with other nutrients,^26–29^ suggesting that protein was not a unique factor limiting their growth.

There are several possible reasons for the lack of effect of additional protein on children’s growth in these studies: (1) initial dietary protein intakes were adequate to meet growth requirements; (2) the supplement displaced usual food intake, causing little or no net increase in protein intake; (3) energy intakes were inadequate despite the provision of the supplement; (4) protein requirements were greater than normal because of the need for catch-up growth due to previous malnutrition or concurrent infections, and the amount of supplemental protein was insufficient to meet this need; or (5) underlying intestinal dysfunction, infections, or systemic inflammation prevented a growth response to the increased protein intake.

The community-based studies reviewed here had little information on the baseline dietary protein or energy intakes of the children. Thus, it is not certain whether the lack of response in those studies was because the children in the study populations already had adequate protein intakes but energy intakes were inadequate or because the supplement simply displaced part of the usual diet, resulting in no net increase in protein intake.

The initial anthropometric or nutritional status of the children in the studies may have influenced the results. In the studies conducted in Peru, children were recovering from acute malnutrition and experiencing accelerated growth; they thus had greater protein needs than nonmalnourished or mildly malnourished children, possibly increasing the likelihood of detecting a growth impact from provision of additional “available” protein. Nevertheless, these studies found that the recommended protein intakes supported the expected growth rates for children of similar age and body size, suggesting that they also would be adequate for nonmalnourished children. In the Malawi studies, many of the children were already stunted or moderately stunted when enrolled at 6 months^25^ or 6–17 months^22^ of age, which may indicate other contextual factors limiting the children’s growth, such as an earlier nutritional insult occurring during pregnancy or preconception, and it is unclear how this may affect the ability of the infant to grow normally or respond to nutritional interventions during early childhood.^2^

**Figure 1:**
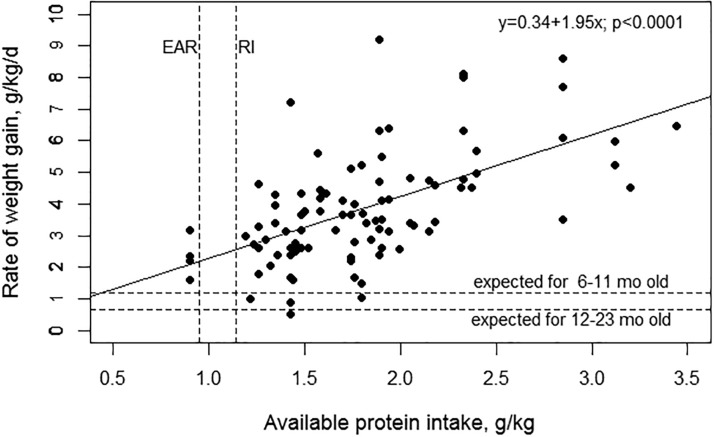
**Association between rate of weight gain and available protein intake in Peruvian children aged 6–32 months recovering from acute malnutrition.** Available protein refers to the total protein intake adjusted for the protein-digestibility amino acid score (PDCAAS). The estimated average requirement (EAR) and recommended intake (RI) of protein (2 standard deviations above EAR) for a child aged 1 year are depicted by vertical lines, and the expected rates of weight gain are depicted in horizontal lines. The figure includes 92 data points, 86 representing individual children from 5 studies [Graham et al. (1969)^10^; Graham et al. (1990)^14^; Lopez de Romaña et al. (1981)^15^; MacLean et al. (1979)^16^; and Graham et al. (1996)^17^] and 6 representing group means [Graham et al. (1986)^13^] because data on individual children were not available.

To further examine relationships between protein intake and growth, estimates of available energy and protein intakes and rate of weight gain per kilogram BW were compiled from the 6 studies by Graham et al. in which the data were available or could be estimated.^10^^,^^13–17^ These 6 studies provided information on 122 children, all of whom were recovering from acute malnutrition and were gaining weight more rapidly than expected for age. A total of 92 data points were included in a graph (Figure 1), with 86 points representing individual children and 6 points representing group means due to unavailable data on individual children. In all cases, weight gains exceeded the average expected rates for children aged 6–12 months (1.2 g/kg BW/d) and 12–24 months (0.7 g/kg BW/d), according to the WHO growth reference values, indicating that even with the lowest levels of available protein the children were able to achieve the expected rate of weight gain for age. At the observed accelerated rates of weight gain, available protein intake was positively associated with weight gain. In a linear regression model, a 1 g increase in available protein per kilogram BW was associated with a 1.94 g/kg BW greater weight gain (*P* < 0.0001; *R*^2 ^= 0.34). The association was only slightly attenuated by including energy intake (per kilogram BW) in the model; the β-coefficient for available protein was 1.30 (*P* < 0.0001; *R*^2 ^= 0.38). In most cases, the amount of available protein provided to the children exceeded the recommended intake level (1.0–1.1 g/kg BW for a 1-year-old). The highest rates of weight gain were achieved with cow’s milk formula and with wheat-based diets with at least 0.2% lysine enrichment, which resulted in a similar rate of gain as when children were fed casein. These diets provided at least 1.4 g of available protein per kilogram BW. There is some uncertainty about the protein needs for catch-up growth due to wide variation in the rates and composition of weight gain and uncertainties about the actual efficiency of protein utilization.^7^ Nevertheless, improvements in the protein quality of grains appear to support accelerated growth if sufficient quantities of both protein and energy are provided.

Few of the community-based studies reviewed reported morbidity information of the children. The interactions between protein, infection, and growth can be viewed in 2 ways: diarrhea or other infections could negatively affect the utilization of protein and result in reduced growth, or adequate protein intake could reduce morbidity or gut dysfunction and positively affect growth. The glutamine study reported no effect of supplementary glutamine on diarrhea prevalence or growth.^23^ The third QPM trial in Ghana reported that children who consumed QPM had significantly fewer days with illness (although the type of illness was not specified) and greater height gains than the children who consumed CM.^20^ A lysine supplementation trial in Ghana, which was not included in the review because of the older age of the children (mean age, 8 years), reported significantly lower rates of diarrhea with lysine supplementation but nonsignificant improvements in growth indices over the 16-week trial.^28^ The impact of lysine and other amino acids on diarrhea in younger children, who typically experience higher diarrhea prevalence and gut dysfunction than school-age children, should be studied further. Also, infections increase nitrogen losses, and it is estimated that protein requirements increase by as much as 20%–25% due to infections^31–33^; however, data in young children regarding the additional protein needs during infection, particularly with chronic or recurrent subclinical infections commonly seen in low-income countries, are lacking.

## CONCLUSION

In summary, although the community-based studies reviewed here did not consistently indicate that providing additional protein or higher quality protein increased children’s growth, the available studies were suboptimal with regard to critical design issues that are necessary to determine whether dietary protein inadequacy is an important cause of growth restriction. The community-based studies did not quantify total protein intakes, including breast milk, or the quality of protein in the diets prior to the intervention, nor did they assess biomarkers of protein status. It was, therefore, unclear whether protein intake or protein utilization was inadequate. Although the results of some of the QPM trials suggested an increase in growth, the results were inconsistent, and the poor quality of the studies undermines their utility.

In order to examine whether additional protein may improve growth in children at risk for growth faltering, the following criteria are recommended for future studies. Studies should target children who are in the age range in which growth faltering typically starts and when complementary foods are introduced (6–12 mo) and who reside in communities where the risk of stunting during the first 2 years is high. Prior research in the community should confirm that children in the target age range have low or marginal intakes of available protein in relation to current estimated requirements or some other marker of low protein status. The intervention should provide a sufficient quantity of protein to bring total available protein intakes up to and above the estimated requirements, considering the increased need for protein during and after infections. The study should also ensure that sufficient energy is provided so that the supplementary protein can be utilized for growth rather than potentially being diverted to meet maintenance energy needs. The intervention must be delivered for an adequate duration to assess linear growth. The sample size should also be large enough to permit examination of potential effect modifiers, such as diet, initial anthropometric status, and morbidity, to identify the population subgroups most in need of future intervention.

## Supplementary Material

Supplementary table

## Supporting Information

The following Supporting Information is available through the online version of this article at the publisher’s website.

*Table S1* **Calculation of protein digestibility-corrected amino acid score (PDCAAS) for single foods**

*Table S2* **Example calculation of protein digestibility-corrected amino acid score (PDCAAS) for mixed diet**
