# Supplementary material for: Effects of protein or amino-acid supplementation on the physical growth of young children in low-income countries
Source: Nutr Rev. 2017 Jul 29;75(9):699–717. doi: 10.1093/nutrit/nux027 (PMC5914345; doi:10.1093/nutrit/nux027)
Supplement: Supplementary table [file nux027_supp.docx]

Supplementary Table 1. Calculation of Protein Digestibility-Corrected Amino Acid Score (PDCAAS) for single foods

|  | Rice | Wheat | Sorghum | Maize | Lentils | Milk |
| --- | --- | --- | --- | --- | --- | --- |
| USDA code ^1^ | 20450 | 20630 | 20648 | 20320 | 16069 | 01077 |
| Protein, g/100 g | 6.6 | 9.7 | 8.4 | 8.1 | 24.6 | 24.6 |
| Amino acids, mg/g protein of food |  |  |  |  |  |  |
| Lysine | 36 | 27 | 21 | 28 | 70 | 84 |
| Sulfur-AA | 44 | 41 | 37 | 39 | 22 | 32 |
| Threonine | 36 | 27 | 37 | 38 | 36 | 43 |
| Tryptophan | 12 | 11.6 | 12.6 | 7.0 | 9.0 | 12.7 |
|  |  |  |  |  |  |  |
| Ratio of amino acid/g protein in food to reference protein scoring pattern ^2^ | | | | | | |
| Lysine | 0.63 | 0.47 | 0.36 | 0.49 | 1.23 | 1.47 |
| Sulfur-AA | 1.62 | 1.50 | 1.37 | 1.44 | 0.80 | 1.20 |
| Threonine | 1.15 | 0.88 | 1.20 | 1.21 | 1.16 | 1.37 |
| Tryptophan | 1.37 | 1.37 | 1.48 | 0.83 | 1.06 | 1.40 |
|  |  |  |  |  |  |  |
| Amino Acid Score | 0.63 | 0.47 | 0.36 | 0.49 | 0.80 | 1.00^3^ |
| Digestibility factor ^4^ | 0.88 | 0.96 | 0.74 | 0.85 | 0.78 | 0.95 |
|  |  |  |  |  |  |  |
| PDCAAS (lowest ratio × digestibility factor) | 0.56 | 0.45 | 0.27 | 0.42 | 0.62 | 0.95 |

^1^ Source of information on protein and amino composition of foods is *USDA Standard Reference Version 28.*

^2^ Reference amino acid pattern for young children (57 mg lysine/g protein, 27 mg sulfur amino acids/g protein, 31 mg threonine/g protein, 8.5 mg tryptophan/g protein) from *FAO. Dietary protein quality evaluation in human nutrition. Report of an FAO Expert Consultation. FAO Food and Nutrition Paper 92. Rome: Food and Agricultural Organization of the United Nations, 2013.*

^3^ Truncation of amino acid score is recommended by *WHO/FAO/UNU. Protein and amino acid requirements in Human Nutrition. Report of a Joint WHO/FAO/UNU Expert Consultation. WHO Technical Report Series 935. Geneva: World Health Organization, 2007*.

^4^ Digestibility factors from *FAO. Protein quality evaluation in human nutrition. Report of Joint FAO/WHO Expert Consultation 4-8 December 1989. FAO Food and Nutrition Paper 51. Rome: Food and Agriculture Organization of the United Nations, 1991.*

Supplementary Table 2. Example calculation of Protein Digestibility-Corrected Amino Acid Score (PDCAAS) for mixed diet ^1^

| Food item | Grams consumed | Protein, g/100 g food ^2^ | Amino acid content of food, mg/g protein ^2^ | | | |  | Protein consumed, g | | | Digestibility factor ^3^ | |  | Digestible protein, g | Digestible amino acid , mg | | | | | |
| --- | --- | --- | --- | --- | --- | --- | --- | --- | --- | --- | --- | --- | --- | --- | --- | --- | --- | --- | --- | --- |
|  |  |  |  |  |  |  | |  |  | |  | |  | (G x H) | C × I | D × I | E × I | | F × I | |
|  | A | B | C | D | E | F | |  | G | | H | |  | I | J | K | L | | M | |
|  |  |  | Lys | SAA | Thr | Trp | |  |  | |  | |  |  | Lys | SAA | Thr | | Trp | |
| Milk | 150 | 3.2 | 84 | 32 | 43 | 13 | |  | 4.8 | | 0.95 | |  | 4.6 | 383 | 146 | 196 | | 59 | |
| Maize | 50 | 8.1 | 28 | 39 | 38 | 7 | |  | 4.1 | | 0.85 | |  | 3.4 | 97 | 134 | 130 | | 24 | |
| Rice | 100 | 6.6 | 36 | 44 | 36 | 12 | |  | 6.6 | | 0.88 | |  | 5.8 | 210 | 255 | 208 | | 68 | |
|  |  |  |  |  |  |  | |  |  | |  | |  |  |  |  |  | |  | |
| Weighted average digestibility (sum of digestible protein/total protein) | | | | | | | | | | | 0.89 | |  |  |  |  |  | |  | |
|  | | | | | | | | | | | | | |  |  |  |  | |  | |
| Total: | | | | | | | | | | | | | | 13.8 | 690 | 535 | 533 | | 151 | |
|  |  |  |  |  |  |  | |  |  | |  | |  |  |  |  |  | |  | |
|  | | | | | | | | | | |  | |  |  |  |  |  | |  | |
| Amino acid mg/g protein (total of each digested aa/total digestible protein) | | | | | | | | | | |  | |  |  | 50 | 39 | 39 | | 11 | |
|  | | | | | | | | | | |  | |  |  |  |  |  | |  | |
|  |  |  |  |  |  |  | |  |  | |  | |  |  |  |  |  | |  | |
| Reference pattern mg/g protein | | | Lys | SAA | Thr | Trp | |  |  | |  | |  |  | Amino acid score: aa mg/g protein divided by mg/g reference protein | | | | | |
|  |  |  | 57 | 27 | 31 | 8.5 | |  |  | |  | |  |  | 0.88 | 1.44 | | 1.25 | 1.29 | |
|  |  |  |  |  |  |  | |  |  | |  | |  |  |  |  | |  |  | |
|  |  |  |  |  |  |  | |  | |  |  |  | |  |  |  | |  |  | |
| PDCAAS (lowest aa score x weighted digestibility factor) | | | | | | | | | | | | | |  | 0.78 |  | |  |  | |
|  |  |  |  |  |  |  | |  | |  |  |  | |  |  |  | |  | |  |

^1^ Adapted from Table 6 in *WHO/FAO/UNU. Protein and amino acid requirements in Human Nutrition. Report of a Joint WHO/FAO/UNU Expert Consultation. WHO Technical Report Series 935. Geneva: World Health Organization, 2007*.

^2^ Source of information on protein and amino composition of foods is *USDA Standard Reference Version 28* (code 01077 milk, 20320 maize, and 20450 rice)*.*

^3^ Digestibility factors from *FAO. Protein quality evaluation in human nutrition. Report of Joint FAO/WHO Expert Consultation 4-8 December 1989. FAO Food and Nutrition Paper 51. Rome: Food and Agriculture Organization of the United Nations, 1991.*
